# Relationships between Bat Swing Speed and Muscle Thickness and Asymmetry in Collegiate Baseball Players

**DOI:** 10.3390/sports5020033

**Published:** 2017-06-02

**Authors:** Ryo Tsuchikane, Takatoshi Higuchi, Tadashi Suga, Michio Wachi, Jun Misaki, Daichi Tanaka, Yuto Miyake, Tadao Isaka

**Affiliations:** 1 Faculty of Sport and Health Science, Ritsumeikan University, 1-1-1 Noji-higashi, Kusatsu, Shiga 525-8577, Japan; sh0060sr@ed.ritsumei.ac.jp (R.T.); t-suga@fc.ritsumei.ac.jp (T.S.); michimichiwachio@yahoo.co.jp (M.W.); sh0044ir@ed.ritsumei.ac.jp (J.M.); sh0058hf@ed.ritsumei.ac.jp (D.T.); sh0067ri@ed.ritsumei.ac.jp (Y.M.); isaka@se.ritsumei.ac.jp (T.I.); 2 Faculty of Socio-Environmental Studies, Fukuoka Institute of Technology, 3-30-1 Wajiro-higashi, Higashiku, Fukuoka 811-0295, Japan; 3 Kanazawa Orthopaedic & Sports Medicine Clinic, 8-8-1 Ono, Ritto, Shiga 520-3016, Japan

**Keywords:** hitting, bat swing speed, ultrasonography, lateral dominance, abdominal muscle, back muscle

## Abstract

The purpose of the present study was to examine the relationships between bat swing speed (BSS), muscle thickness, and muscle thickness asymmetry in collegiate baseball players. Twenty-four collegiate baseball players participated in this study. Maximum BSS in hitting a teed ball was measured using a motion capture system. The muscle thicknesses of the trunk (upper abdominal rectus, central abdominal rectus, lower abdominal rectus, abdominal wall, and multifidus lumborum), upper limb, and lower limb were measured using a B-mode ultrasonography. Lateral asymmetry between each pair of muscles was determined as the ratio of the thickness of the dominant side to that of the non-dominant side. Statistically significant positive correlations were observed between BSS and muscle thicknesses of the abdominal wall and multifidus lumborum on the dominant side (*r* = 0.426 and 0.431, respectively; *p* < 0.05), whereas only trends against this significance were observed between BSS and muscle thicknesses on the non-dominant side. No statistical correlations were found between BSS and the lateral asymmetry of any muscles. These findings indicate the importance of the trunk muscles for bat swing, and the lack of association between BSS and lateral asymmetry of muscle size.

## 1. Introduction

Baseball hitting requires making contact with a thrown ball that passes the home plate in less than 0.5 s. In addition, a certain batted ball speed is necessary to avoid being caught by field players and to travel farther. Therefore, bat swing speed (BSS) is one of the main determinants of baseball hitting performance, because a higher BSS can produce a shorter swing time and higher batted-ball velocity [1,2]. To develop a training program that effectively improves BSS, key components of the bat swing have been investigated. Studies of high school and collegiate baseball players have revealed statistically significant positive correlations between BSS and maximum muscle strength of the upper and lower limbs [3,4,5]. Therefore, because muscle volume and muscle strength are closely related, muscle volume also should be positively correlated with BSS [6]. Unlike muscle strength measured by dynamometers, muscle size can be measured precisely independent of synergistic muscles using a clinical imaging apparatus, such as ultrasonography, computed tomography, or magnetic resonance imaging. Recently, the relationship between muscle size and athletic performance was revealed [7]. The measurement of individual trunk muscle size can be used to determine the prime mover muscle to generate higher BSS. However, to the best of our knowledge, there is no report on the relationship between muscle size and BSS in baseball players.

Ultrasonography is a practical method to estimate muscle size because muscle thickness measured using ultrasonography statistically significantly correlates with muscle cross-sectional area and volume measured using computed tomography and magnetic resonance imaging [8,9]. Moreover, the price and running cost of the ultrasonography are lower than other methods. In addition, the accessibility of ultrasonography allows frequent monitoring of muscle thickness, which can be useful for the assessment of training effect on target muscles. Therefore, ultrasonography may be suitable for assessing athletes’ potential performance, once the relationship between muscle thickness and the performance variable is clarified.

The motion of baseball hitting consists of coordinative actions of multiple body segments, including trunk rotation [10]. Trunk muscles play essential roles in posture control and trunk rotation [11,12]. A previous electromyographic study confirmed the contribution of trunk muscle activity to generate a large amount of force to accelerate the bat during the initial phase of bat swing [13]. Therefore, trunk muscle training for baseball hitting should consist of coordinative multi-segment motion with high force production in a short amount of time. Previous research has shown that training by throwing a medicine ball using trunk rotation improved BSS in high school baseball players [14]. Thus, both motor competence to generate rotational force and trunk muscle size in baseball players may be related to the maximum BSS. Although a comparison of hitting kinematics among different ages and competition levels was conducted in previous studies [15], the association between the trunk muscle size and BSS has not been investigated.

Although abdominal muscles and back muscles have been considered as agonists for trunk rotation, more detailed information such as the timing and location of the specific muscles which contribute to the bat swing is unknown. Shaffer et al. [13] reported a lateral difference in trunk muscle activity during baseball hitting. Lateral asymmetry of the trunk muscles has been shown to exist in athletes of sports with unilateral dominance, such as soccer and tennis [16]. Because of dominant handedness and repeated unidirectional rotary movement during baseball hitting and throwing, lateral asymmetry of the trunk muscles also may exist in baseball players. However, the influence of such asymmetry on BSS is unknown. If such asymmetry is not related to superior athletic performance, it should be avoided. The purpose of the present study was to examine the relationship between BSS and muscle thickness. Additionally, we examined the relationship between lateral asymmetry in these muscles and BSS.

## 2. Materials and Methods

### 2.1. Study Design

The BSS of each subject during tee-ball hitting was used to represent their ability to produce the maximal BSS. Muscle thickness was measured as an indicative parameter for muscle size. A cross-sectional design was employed to analyze the correlations between BSS, muscle thickness, and lateral asymmetry of the trunk and limbs in order to examine the relationships of baseball hitting performance with muscle thickness and lateral asymmetry.

### 2.2. Participants

Twenty-four collegiate baseball players (age: 20.5 ± 0.7 years, height: 1.74 ± 0.04 m, body mass: 71.4 ± 5.9 kg) participated in this study. All baseball players in this study were position players with a duration of baseball experience of at least 12 years (mean: 13.5 ± 1.4 years). Their team is a member of the Kansai BIG 6 Baseball League in Japan, which is a division I collegiate baseball league. Eleven players used their right hand for swinging, whereas 13 players used their left hand for swinging. The measurement was conducted in December, when the team finished the season and started the winter training period. This research protocol was approved by the university institutional ethical review board, and was conducted in accordance with the Declaration of Helsinki. Written informed consent was obtained from all participants after explaining the experimental procedures, risks, and benefits.

### 2.3. Procedures

In accordance with a previous baseball hitting study [17], first, participants conducted a warm-up consisting of stretching, calisthenics, and dry swings. Then they performed practice hitting of a tee-ball with submaximal and maximal efforts. After the warm-up and practice hitting, participants completed five hits of a tee-ball toward a target set about 6 m away from the hitter in the center field direction using their maximum effort. Sixteen 500-Hz infrared cameras (Raptor; NAC Image Technology Inc., Tokyo, Japan) were utilized to capture the movement of a reflective marker attached on the barrel end of a wood bat. The length and mass of the bat were 0.84 m and 0.9 kg, respectively. Motion analysis software (Cortex 4.0; Motion Analysis, Santa Rosa, CA, USA) was used to track and analyze each trial. The measurement space in which bat swing was performed was calibrated using a dynamic wand calibration method with a wand kit. BSS was the magnitude of the resultant velocity during the 10 ms immediately prior to the moment of ball-bat contact, which was detected using a sound-to-electrical transducer trigger unit (ATRG-100; Nihon Fastec Imaging Co. Ltd., Tokyo, Japan). The mean of the three highest BSSs of the five trials represented each participant’s BSS.

A B-mode ultrasonographic device (SSD-3500SV; Aloka, Tokyo, Japan) using a linear scanner with a sampling rate of 7.5 MHz was utilized to measure the thickness of the muscles of the trunk (upper abdominal rectus, central abdominal rectus, lower abdominal rectus, abdominal wall, and multifidus lumborum), upper limbs (elbow extensors, elbow flexors, and forearm muscles), and lower limbs (knee extensors, knee flexors, ankle dorsiflexors, and ankle plantar flexors) on both sides. Thicknesses of the upper rectus abdominis and central rectus abdominis were measured at the second and third layers from the proximal fibrous band to the intermediate fibrous band, respectively. Thickness of the lower rectus abdominis was measured at the fourth and most distal layer from the umbilical fibrous band to the pubic area. In the lateral abdominal wall, muscle thickness was separated into external abdominal oblique, internal abdominal oblique, and transverse abdominal oblique. Thicknesses of the external abdominal oblique, internal abdominal oblique, and transverse abdominal oblique were measured at 15 mm from the muscle tendon junction of the transverse abdominal oblique in the muscle belly of the lateral abdominal wall [18]. Thickness of the multifidus lumborum was measured at the spinous process of the L5 vertebra [19]. The measured limb muscles were as follows: elbow flexor, elbow extensor, forearm flexor, knee flexor, knee extensor, dorsiflexor, and plantar flexor. Thicknesses of the limb muscles were measured based on the method used in previous studies [20]. In brief, thicknesses of the elbow flexor and elbow extensor were measured at 60% of upper arm length (the distance from the acromion process of the scapular to the lateral epicondyle of the humerus). Thickness of the forearm flexor was measured at 30% of forearm length (the distance from the styloid process and the head of the radius). Thicknesses of the knee flexor and knee extensor were measured at 50% of thigh length (the distance from the greater trochanter of the femur to the articular cleft between the femur and tibia condyles). Thicknesses of the dorsiflexor and plantar flexor were measured at 30% of lower leg length (the distance from the articular cleft between the femur and tibia condyles to the lateral malleolus). Lateral asymmetry of muscle thickness for each participant was calculated by the equation below:

(1) $$\mathrm{Asymmetry}\;\left(\%\right)=\;\frac{\mathrm{dominant}\;\mathrm{side}\;\mathrm{thickness}}{\;\mathrm{non}-\mathrm{dominant}\;\mathrm{side}\;\mathrm{thickness}}\;\times \;100$$

in which, for a right-handed hitter, the dominant side was the right side and the non-dominant side was the left side. Although the reliability of thicknesses of the limb muscles has been frequently examined [8], the reliability of thicknesses of the trunk muscles has been poorly examined. Therefore, in a recent study [21], we examined the intraclass correlation coefficients in thicknesses of the trunk muscles on two separate days in 12 healthy men (age: 22.5 ± 1.6 years, height: 1.70 ± 0.03 m, weight: 63.8 ± 6.4 kg). Accordingly, the intraclass correlation coefficients for the right and left sides were 0.960 for right side and 0.965 for left side in the upper abdominal rectus, 0.963 for right side and 0.959 for left side in the central abdominal rectus, 0.959 for right side and 0.946 for left side in the lower abdominal rectus, 0.970 for right side and 0.921 for left side in the abdominal wall, and 0.919 for right side and 0.965 for left side in the multifidus lumborum.

### 2.4. Statistical Analyses

Data were expressed as mean ± SD. The Pearson product-moment correlation (*r*) between BSS and muscle thickness of the right and left sides and lateral asymmetry between of the bilateral sides were calculated and used to analyze the relationship. A paired t-test was used to compare muscle thickness between the dominant and non-dominant sides. All statistical analyses were conducted using SPSS, version 19.0 (IBM Corp., Armonk, NY, USA), with the level for statistical significance set at *p* < 0.05.

## 3. Results

The mean BSS was 34.3 ± 2.4 m·s^−1^. The mean (±standard deviation, SD) value of the coefficient of variation for the three highest BSSs of each participant was less than 1.2% (±0.8%). Muscle thickness of the dominant and non-dominant sides and the rate of lateral asymmetry are presented in Table 1. The abdominal wall and multifidus lumborum were significantly larger in the non-dominant side than in the dominant side.

**Figure 1:**
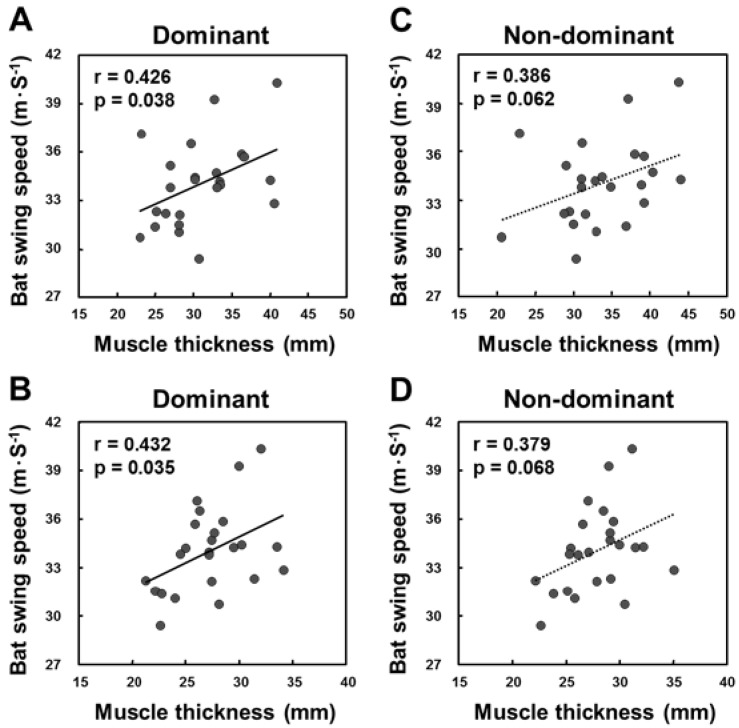
Scatterplots with regression lines for bat swing speed (m·s^−1^) and muscle thickness (mm) of the abdominal wall on the dominant side (**A**), the multifidus lumborum on the dominant side (**B**), the abdominal wall on the non-dominant side (**C**), and the multifidus lumborum on the non-dominant side (**D**).

A matrix for the correlation coefficients between BSS and muscle thickness of the dominant and non-dominant sides and the rate of lateral asymmetry is presented in Table 2. Statistically significant positive correlations were found between BSS and muscle thickness of the abdominal wall and multifidus lumborum on the dominant side (Figure 1A,B). While the moderate effect size was observed between BSS and the muscle thickness of the abdominal wall and multifidus lumborum on the non-dominant side (Figure 1C,D), and elbow flexors on the dominant side, no correlation but rather a tendency against this was obtained. By contrast, no statistically significant correlations were found between BSS and the lateral asymmetry of the measured muscles.

## 4. Discussion

The aim of the present study was to examine the relationships between BSS, muscle thickness, and lateral asymmetry of the trunk and limbs in collegiate baseball players. Based on their BSSs, the level of the players was about the same as that of other collegiate baseball players who participated in a previous study [22]. BSS statistically significantly correlated with only muscle thickness of the abdominal wall and multifidus lumborum on the dominant side; other trunk muscles, upper and lower limb muscles, and lateral asymmetry were not statistically significantly correlated. These results suggest that the trunk muscles, especially the abdominal wall and multifidus lumborum on the dominant side, may be important for higher BSS in baseball players. Since the abdominal walls and multifidus longus on the dominant and non-dominant sides were moderately correlated to BSS (Figure 1), both sides of muscle thickness may be related to BSS.

In the present study, muscle thickness of the abdominal wall and multifidus lumborum on the dominant side positively correlated with BSS. The abdominal wall is highly active in trunk rotation [23]. High electromyographic activity of the abdominal wall was present from the pre-swing or loading phase to the follow-through phase of the hitting motion in 18 professional baseball players [13]. In a similar way, the multifidus lumborum serves as a trunk rotator [24,25]. Therefore, the large size of the abdominal wall and multifidus lumborum may contribute to producing a greater rotational force of the trunk, thereby contributing to the generation of higher BSS. On the other hand, muscle thickness of the abdominal wall and multifidus lumborum on the non-dominant side demonstrated a smaller correlation with BSS. Similarly, Shaffer and colleagues [13] reported that electromyographic activity of the abdominal wall on the non-dominant side was lower than that on the dominant side. However, further investigation of the kinetic and kinematic aspects of the trunk muscles during the hitting motion is necessary to clarify any causal associations.

Muscle thickness of the upper and lower limbs did not statistically significantly correlate with BSS in the present study (Table 2). However, the relationship between BSS and muscle strength of the upper and lower limbs seems to vary depending on their maturity in strength and bat swing skill. Previous studies have found statistically significant correlations between BSS and muscle strength of the upper and lower limbs in high school baseball players [3,14], whereas collegiate baseball players have shown inconsistent results regarding the correlation between BSS and upper and lower limb muscle strength [2]. The findings of the present study suggest that muscle thickness of the upper and lower limbs does not correlate with BSS. In previous studies, resistance training emphasizing the upper and lower limbs effectively increased BSS in high school baseball players but not in collegiate baseball players [14,23]. Such difference between high school and collegiate players can be explained by the higher trainability of high school players because of their less developed muscle strength and BSS. In a previous study of high school baseball players, Miyaguchi and colleagues [3] reported a statistically significant correlation between the bat swing velocity and maximum load for bench press with one repetition in a group of players who had hit a home run in their league. In contrast, they also found no correlation between the bat swing velocity and maximum load for bench press with one repetition in a group of players who had never hit a home run. Based on these studies, improvement in muscle strength of the whole body, including the upper and lower limbs, may be crucial to improve swing mechanics and BSS in poorly-trained hitters. On the other hand, improvement in only the trunk muscles, which are responsible for trunk rotation, could effectively increase BSS in well-trained hitters. In fact, in a previous study, a training protocol including maximum trunk rotation with a bat swing–like posture successfully increased bat swing velocity in collegiate baseball players [17]. Therefore, the hitter’s strength level, specificity of training for baseball hitting, and BSS should be considered in designing an effective training program for improving the muscle strength of the upper and lower limbs.

Lateral asymmetry, which can be harmful because of its mechanical strain on the body, is common for athletes in sports with repetitive throwing and striking [13]. For example, lateral asymmetry is a well-known consequence of playing soccer and tennis [16]. In baseball, the unilateral dominance of throwing and hitting motions is considered to be the cause of apparent lateral asymmetry of muscle size, strength, and flexibility [26]. However, there has been no study on the relationship between baseball hitting performance and lateral asymmetry. The present study found no correlation between BSS and lateral asymmetry of muscle thickness. The results in the present study suggest that muscle size rather than lateral asymmetry is more important to achieve high BSS. Because lateral asymmetry of trunk muscle size can be associated with injury and back pain [27], equalizing such asymmetry is beneficial for hitters by preventing or alleviating related injuries without compromising the potential to produce high BSS.

The positive correlation between BSS and the muscle size of trunk muscle groups which was found in the present study is limited to collegiate baseball players. Therefore, further investigation of the relationship in younger baseball players will clarify the contribution of trunk muscle development to baseball hitting performance over a longer time span. Moreover, the influence of trunk muscle size on the kinematics of the bat swing needs to be clarified to elucidate the mechanism for higher BSS in hitters with greater trunk muscle size. For instance, Thomas et al. [28] reported the adaptation of the neuromuscular component in division I collegiate baseball pitchers to achieve greater performance. Therefore, a hitter’s ability in coordinative movement should also contribute to achieving greater BSS in addition to the trunk muscle size.

## 5. Conclusions

In conclusion, the relationships between BSS, muscle thickness, and lateral asymmetry of the trunk and limbs in collegiate baseball players were examined in the present study. BSS statistically significantly correlated with only muscle thickness of the abdominal wall and multifidus lumborum on the dominant side. These findings indicate, in accordance with previous studies [13], the importance of the trunk muscles for producing high BSS. The present study is the first to find the correlation between BSS and trunk muscle size, which supports the importance of trunk muscle training.

## Figures and Tables

**Table 1:** Muscle thickness (mean ± SD) in dominant and non-dominant sides and asymmetry.

Muscle	Dominant (mm)	Non-Dominant (mm)	Asymmetry (%)
Trunk muscles			
Upper abdominal rectus	16.2 ± 2.4	16.2 ± 2.4	0.0 ± 6.0
Central abdominal rectus	17.4 ± 2.5	17.2 ± 2.6	+1.9 ± 7.0
Lower abdominal rectus	19.5 ± 3.3	19.8 ± 3.5	−0.6 ± 9.9
Abdominal wall	30.9 ± 5.2	33.7 ± 5.9 ^∗^	−7.5 ± 8.6
Multifidus lumborum	27.3 ± 3.5	27.9 ± 3.1 ^∗^	−2.2 ± 4.5
Upper limb muscles			
Elbow flexors	32.3 ± 2.9	32.1 ± 2.9	+1.0 ± 6.1
Elbow extensors	35.1 ± 5.5	35.3 ± 5.6	+0.1 ± 11.2
Forearm flexors	24.5 ± 2.7	24.5 ± 2.5	+0.6 ± 10.1
Lower limb muscles			
Knee extensors	61.7 ± 5.2	60.2 ± 5.4	+2.7 ± 5.9
Knee flexors	76.6 ± 6.6	76.6 ± 5.9	+0.1 ± 3.3
Dorsiflexors	29.5 ± 2.3	29.2 ± 2.3	+1.3 ± 4.0
Plantar flexors	69.4 ± 4.9	69.6 ± 4.5	−0.3 ± 4.5

* Statistically significant difference (*p* < 0.05) between dominant and non-dominant sides.

**Table 2:** Correlation coefficients (*r*) between bat swing speed and muscle thickness of the dominant and non-dominant sides and rate of lateral asymmetry.

Muscle	Dominant		Non-dominant		% Asymmetry	
	*r*	*p* Values	*r*	*p* Values	*r*	*p* Values
Trunk						
Upper abdominal rectus	0.229	0.283	0.151	0.480	0.160	0.456
Central abdominal rectus	0.236	0.267	0.184	0.390	0.077	0.722
Lower abdominal rectus	0.097	0.651	0.098	0.650	0.006	0.978
Abdominal wall	0.426	0.038	0.386	0.062	0.008	0.972
Multifidus lumborum	0.432	0.035	0.379	0.068	0.261	0.218
Upper limb						
Elbow flexors	0.378	0.069	0.223	0.295	0.183	0.393
Elbow extensors	−0.149	0.487	−0.015	0.945	−0.201	0.346
Forearm flexors	0.003	0.989	−0.143	0.505	0.167	0.436
Lower limb						
Knee extensors	0.194	0.364	0.245	0.249	−0.105	0.624
Knee flexors	0.081	0.706	0.028	0.898	0.136	0.528
Dorsiflexors	−0.110	0.609	0.005	0.980	−0.237	0.265
Plantar flexors	−0.044	0.837	0.152	0.478	−0.286	0.176
